# An Elevated Platelet-to-Lymphocyte Ratio Predicts Poor Prognosis and Clinicopathological Characteristics in Patients with Colorectal Cancer: A Meta-Analysis

**DOI:** 10.1155/2017/1053125

**Published:** 2017-04-26

**Authors:** Xuan-zhang Huang, Wen-jun Chen, Xi Zhang, Cong-cong Wu, Chao-ying Zhang, Shuang-shuang Sun, Jian Wu

**Affiliations:** Department of Chemotherapy and Radiotherapy, The Second Affiliated Hospital and Yuying Children's Hospital of Wenzhou Medical University, 109 Xueyuan West Road, Lucheng District, Wenzhou 325027, China

## Abstract

*Background.* The aims of this study were to evaluate the clinicopathological and prognostic values of platelet-to-lymphocyte ratio (PLR) in colorectal cancer (CRC). *Methods.* The PubMed and Embase databases and the references of relevant studies were systematically searched. This study was performed with hazard ratios (HRs) and odd ratios (ORs) with corresponding 95% confidence intervals (CIs) as effect measures. *Results.* Our results indicated that elevated PLR was associated with poor overall survival (HR = 1.46, 95% CI = 1.23–1.73), disease-free survival (HR = 1.64, 95% CI = 1.17–2.30), cancer-specific survival (HR = 1.30, 95% CI = 1.12–1.51), and recurrence-free survival (HR = 1.38, 95% CI = 1.09–1.74) in CRC. For the clinicopathological characteristics, our results indicated that there were differences in the rate of elevated PLR between stages III/IV and I/II groups (OR = 1.38, 95% CI = 1.01–1.88), pT3/T4 and pT1/T2 groups (OR = 1.82, 95% CI = 1.03–3.20), and poor differentiation and moderate/well differentiation (OR = 2.59, 95% CI = 1.38–4.84). *Conclusions.* Our results indicated that elevated PLR predicted poor prognosis and clinicopathological characteristics in CRC and PLR is a convenient and low-cost blood-derived prognostic marker for CRC.

## 1. Introduction

Colorectal cancer (CRC) is the third most frequently diagnosed cancer in males and the second in females [1]. Tumor metastasis and recurrence still remain the major cause of mortality. However, there is a lack of precise biomarkers for predicting prognosis in CRC that can be used for individualized treatment. Thus, it is clinically important to find reliable prognostic markers for cancer treatment.

Bodies of evidence have shown that the interactions between tumor and host-derived microenvironments, such as inflammation, immune response, and coagulation status, play an important role in tumor progression and prognosis [2–4]. Severe inflammatory responses could result in an imbalance of the immune response, promoting tumor progression [3–5]. Recently, as a convenient and cost-effective blood-derived marker, the platelet-to-lymphocyte ratio (PLR), which takes into account the inflammatory response, immune response, and coagulation status, has been widely investigated as a useful prognostic factor in various solid cancers [6, 7]. However, the prognostic values of PLR in CRC are controversial and have not been confirmed [8–12]. Furthermore, whether the PLR could predict the clinicopathological characteristics of CRC is also unclear.

The purposes of the present study were to use a meta-analysis to quantitatively and comprehensively summarize the clinicopathological and prognostic significance of the PLR in CRC.

## 2. Materials and Methods

### 2.1. Literature Search

PubMed and Embase databases were systematically searched for all relevant studies (up to February 2016). Moreover, the reference lists of all relevant studies and reviews were also manually searched to identify any potentially eligible studies. The following search terms were used: “platelet-to-lymphocyte ratio”, “platelet-lymphocyte ratio”, “platelet to lymphocyte ratio”, “colorectal cancer”, “colon cancer”, and “rectal cancer”.

### 2.2. Eligibility Criteria

Studies were included in our meta-analysis if they met all of the following inclusion criteria: (1) the included patients were diagnosed as CRC, (2) the outcome of interest was the clinicopathological and/or prognostic relationship between PLR and CRC, and (3) the outcome measures of interest could be extracted directly or could be calculated from the published data indirectly. If several duplicated studies based on the same population met the inclusion criteria, only the most informative study was included in our meta-analysis.

### 2.3. Data Extraction and Quality Assessment

Eligible studies were reviewed, and data of interest were extracted by two reviewers, independently. The following data were extracted: first author, publication year, country, population characteristics, tumor clinicopathological characteristics, sampling time, cut-off value, rate of elevated PLR, and prognostic value of PLR (overall survival (OS), disease-free survival (DFS), cancer-specific survival (CSS), and recurrence-free survival (RFS)).

The quality of the included studies was evaluated using the Newcastle-Ottawa Scale (NOS) criteria [13]. In addition, any disagreements on data extraction and/or quality assessment were resolved through comprehensive discussion.

### 2.4. Statistical Analysis

Hazard ratios (HRs) and odds ratios (ORs) with corresponding 95% confidence intervals (CIs) were used as measures to summarize the relationship between PLR and prognosis and between PLR and tumor clinicopathological characteristics, respectively. HRs and 95% CIs were extracted directly, or they were calculated from available data using the methods designed by Tierney et al. [14]. Subgroup analyses were conducted stratified by sampling time, metastatic status, sample size, cut-off value, country, and study quality. We also conducted subgroup analysis based on study analysis type in the primary studies to explore the impact of multivariable and univariable analysis.

The heterogeneity among the studies was assessed using the *I*^2^ statistics and Cochran *Q* test. A random effects model was used to pool measures if substantial heterogeneity existed; otherwise, a fixed effects model was used. A metaregression analysis was conducted to explore potential variables that contributed heterogeneity or dominated results [15]. Begg's and Egger's tests were used to evaluate publication bias, and a trim-and-fill analysis was performed to assess the effect of publication bias if a significant publication bias existed [16].

All statistical analyses were performed using Stata software version 12.0 (Stata Corporation, College Station, TX, USA). A two-sided *P* value < 0.05 was considered statistically significant.

## 3. Results

### 3.1. Study Selection and Study Characteristics

**Figure 1:**
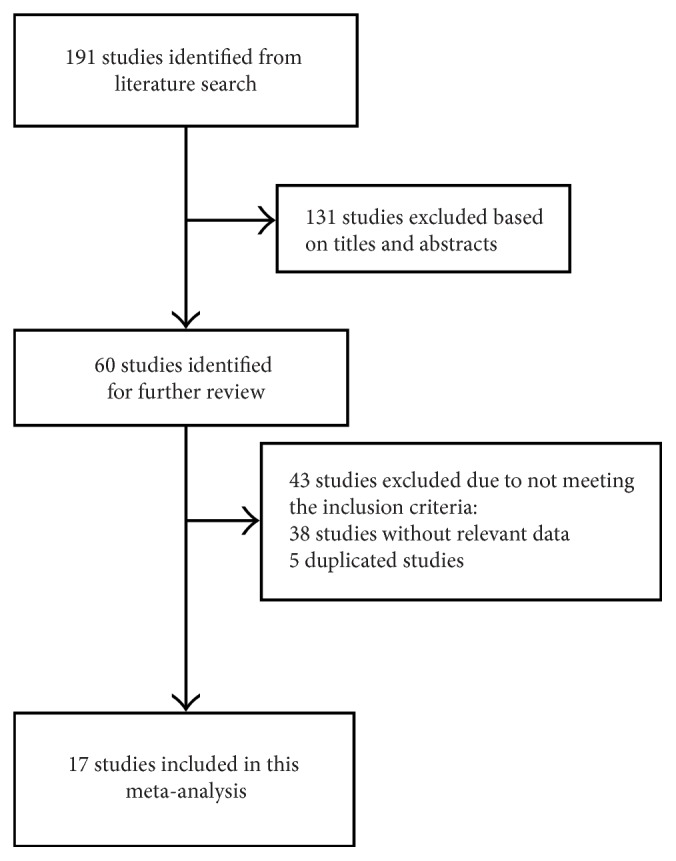
Flow diagram showing the selection process for the included studies.

A total of 191 studies were initially identified from the literature search, and 131 studies were excluded after reviewing the titles and abstracts. After a full-text review, 38 studies were excluded. Finally, 17 studies were included in our meta-analysis (Figure 1) [8–12, 17–28].

The 17 eligible studies included 4968 CRC patients (mean sample size: 292; median and range of sample size: 243 and 110–624). The studies were from the USA, the United Kingdom, Austria, Canada, China, Korea, Hungary, and Japan, and the year of publication ranged from 2011 to 2015. The baseline characteristics and quality of studies are summarized in Table 1.

## 4. Impact of PLR on Survival

### 4.1. PLR and OS

**Figure 2:**
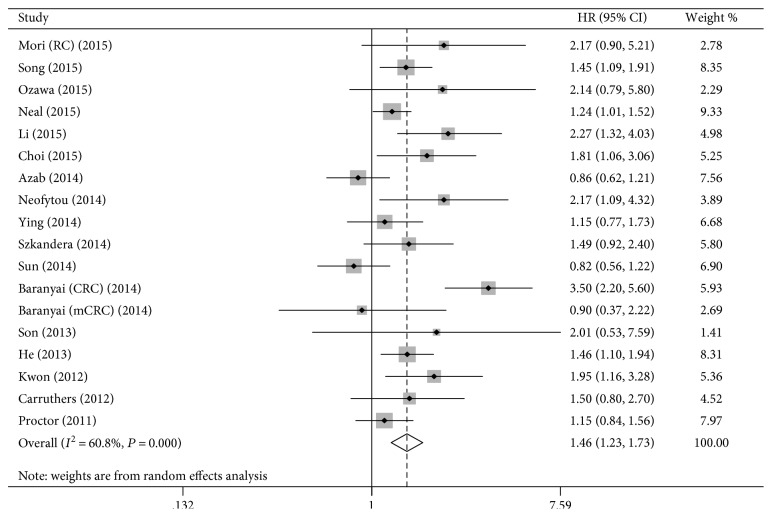
The estimated hazard ratio (HR) was summarized for the relationship between platelet-to-lymphocyte ratio and overall survival.

The pooled estimated HRs indicate that elevated PLR was associated with poor OS in CRC (HR = 1.46, 95% CI = 1.23–1.73, Figure 2). Including studies only assessing preoperative PLR, our results also indicate that elevated PLR predicted a poor OS (HR = 1.61, 95% CI = 1.28–2.02).

We conducted subgroup analyses stratified by cut-off value and sample size, and the results confirmed PLR as a prognostic factor for OS: cut-off value (cut-off > 150: HR = 1.60, 95% CI = 1.18–2.17; cut-off ≤ 150: HR = 1.33, 95% CI = 1.08–1.64) and sample size (sample size ≥ 250: HR = 1.36, 95% CI = 1.01–1.82; sample size < 250: HR = 1.53, 95% CI = 1.32–1.77). As shown by the subgroup analyses stratified by distant metastasis status, study quality, study analysis type, and country, the prognostic effect of PLR on OS was also confirmed (Table 2).

### 4.2. PLR and DFS

**Figure 3:**
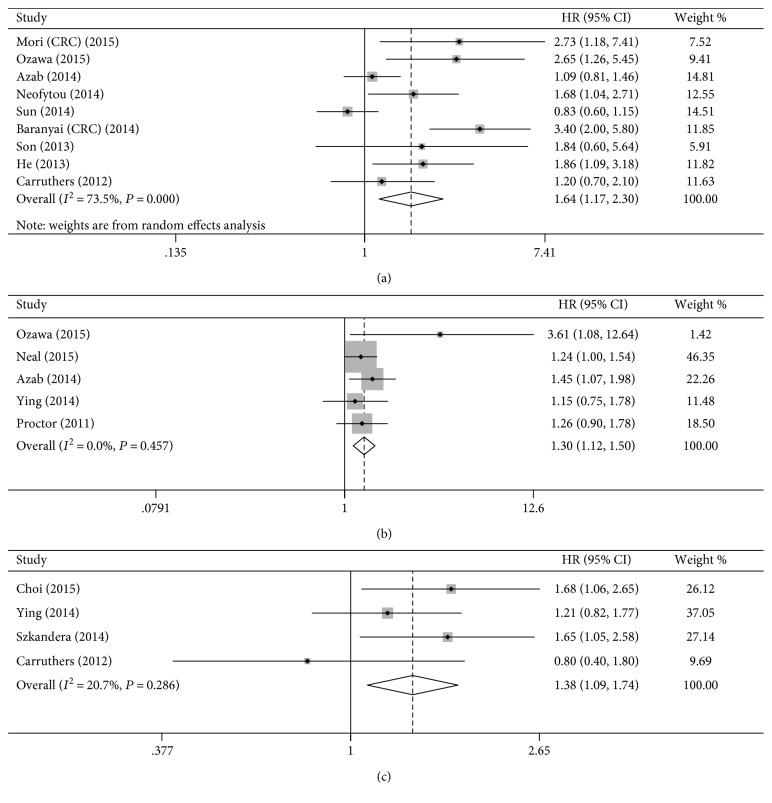
The estimated hazard ratio (HR) was summarized for the relationship between platelet-to-lymphocyte ratio and disease-free survival (a), between platelet-to-lymphocyte ratio and cancer-specific survival (b), and between platelet-to-lymphocyte ratio and recurrence-free survival (c).

The poor prognosis for DFS in CRC was indicated by the elevated PLR (HR = 1.64, 95% CI = 1.17–2.30, Figure 3(a)). Moreover, the result of subgroup analysis for preoperative PLR was similar, predicting poor DFS (HR = 1.78, 95% CI = 1.12–2.83).

The subgroup analysis based on cut-off value ≤ 150 (HR = 1.49, 95% CI = 1.03–2.14) and metastasis positive (HR = 1.76, 95% CI = 1.23–2.51) provided a similar result. In addition, the results of subgroup analyses based on study analysis type, country, and study quality confirmed that elevated PLR tended towards worse DFS (Table 2).

### 4.3. PLR and CSS

Our results indicate that CCS was worse in CRC with elevated PLR compared with those with low PLR (HR = 1.30, 95% CI = 1.12–1.50, Figure 3(b)). Similarly, the results of preoperative PLR showed that elevated PLR was associated with worse CSS (HR = 1.26, 95% CI = 1.04–1.52). We observed a similar result in the subgroup analyses stratified by multivariable analysis type, univariable analysis type, sample size ≥ 250, cut-off value ≤ 150, and NOS ≥ 6 (Table 2).

### 4.4. PLR and RFS

All the studies used a cut-off value > 150 and preoperative PLR. RFS was worse in patients with elevated PLR compared with those with low PLR (HR = 1.38, 95% CI = 1.09–1.74, Figure 3(c)). As was shown by subgroup analyses on metastasis status, sample size, country, and study quality, similar results were observed (Table 2).

## 5. Correlation of PLR with Clinicopathological Characteristics

The meta-analysis of relevant studies on TNM stage indicated a higher rate of elevated PLR in the stage III/IV group relative to the stage I/II group (OR = 1.38, 95% CI = 1.01–1.88), as well as stage II–IV group relative to the stage I group (OR = 2.77, 95% CI = 1.87–4.12). The rate of elevated PLR was different between the pT3/T4 and pT1/T2 groups (OR = 1.82, 95% CI = 1.03–3.20) and poor differentiation and moderate/well differentiation (OR = 2.59, 95% CI = 1.38–4.84). However, we could not observe an association between lymph node metastasis and PLR (OR = 1.16, 95% CI = 0.86–1.57), lymphatic invasion and PLR (OR = 1.48, 95% CI = 0.88–2.46), and venous invasion and PLR (OR = 1.31, 95% CI = 0.79–2.17).

## 6. Publication Bias and Metaregression

**Figure 4:**
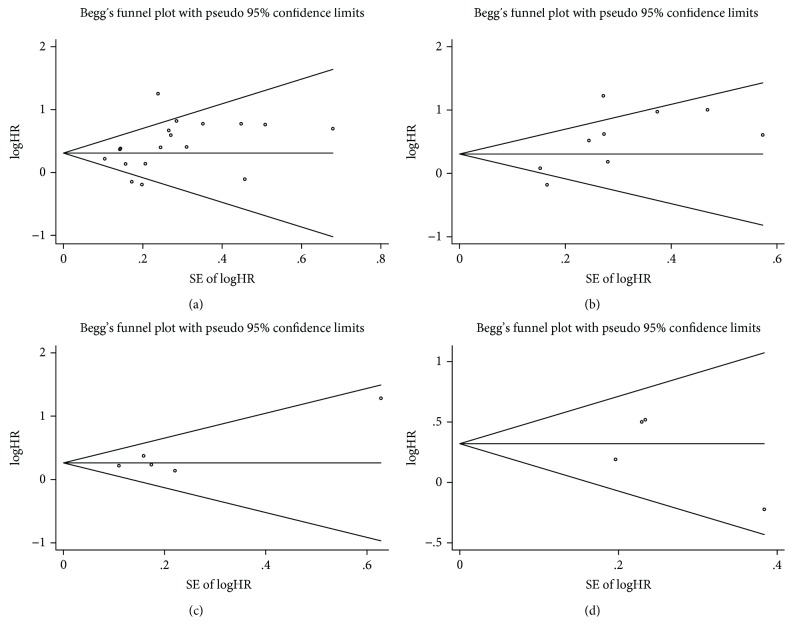
Funnel plots assessing publication bias for overall survival (a), disease-free survival (b), cancer-specific survival (c), and recurrence-free survival (d).

Begg's and Egger's tests showed no substantial publication bias, except in the HRs for DFS. And the funnel plots for analyses are shown in Figure 4. The trim-and-fill analyses indicated that there might be three unpublished or missing studies existing in the meta-analysis of DFS; however, the association between PLR and DFS was still statistically significant even if the three studies were published, indicating that publication bias could not impact on the results for DFS. Our metaregression analysis suggested that sampling time, metastatic status, sample size, cut-off value, country, and study quality were not significant sources of heterogeneity and did not obviously dominate present results (Table 3).

## 7. Discussion

CRC is a global health problem with a high rate of recurrence and metastasis [1]. Thus, there is an urgent need to explore additional prognostic markers to facilitate earlier and optimized treatment for CRC. Recently, many studies have been performed to assess the clinicopathological and prognostic values of PLR in CRC [8–12]. However, to date, there is still no general agreement on the clinical value of PLR in CRC.

Our results indicated that elevated PLR predicted poor survival in CRC, including OS, DFS, CSS, and RFS. In addition, our results also indicated that elevated PLR was associated with poor tumor stage, pT category, and degree of differentiation and suggested that PLR may be feasible for tumor staging in CRC. Similar results were obtained in the subgroup analyses.

Several studies have reported that host-derived inflammation, immune response, and coagulation status played an important role in tumor proliferation, invasion, angiogenesis, and metastasis [2–4]. In cancer, the systemic inflammatory response may be secondary to tumor hypoxia or local tissue damage [29] and resulted in an imbalance of immune response, promoting tumor progression [3–5]. As circulating biomarkers for inflammation, immune response, and coagulation status, platelet and lymphocyte counts were reported to be associated with prognosis in CRC [18, 30]. Thus, we conducted the present study to assess the clinical values of PLR in CRC, and the results indicated that elevated PLR could predict a poor prognosis in CRC.

The underlying mechanisms responsible for the role of PLR in CRC have not yet been elucidated, but recent experimental and clinical data may provide several potential explanations. An elevated PLR represents an increased number of platelets and/or a decreased number of lymphocytes, and elevated platelets could promote metastatic potential of tumor cells in several biological pathways. Platelets could secrete cellular growth factors (i.e., platelet-derived growth factor, vascular endothelial growth factor, transforming growth factor beta, platelet factor 4, and inflammatory mediators) and then stimulate tumor angiogenesis and growth [31, 32]. Besides, several studies have shown that platelets can activate the invasiveness of tumor cells by enhancing the formation of tumor stroma and supporting the stable adhesion of tumor cells to the endothelium [33, 34]. Furthermore, in the bloodstream, the interactions between tumor cells and platelets could facilitate tumor cell metastasis by impeding the clearance of tumor cells by innate immune cells [34, 35]. Thus, many studies were performed to explore the antitumor activity of antiplatelet agents. Indeed, Suzuki et al. reported that antiplatelet drugs (i.e., cilostazol and prostaglandin I2) could inhibit invasiveness of tumor cells [33], and Mikami et al. showed that antiplatelet antibody or aspirin could inhibit proliferation of tumor cells both in vivo and in vitro [36]. Moreover, several clinical trials demonstrated that aspirin use was associated with lower mortality in CRC [37, 38]. Future studies are needed to explore the tailored treatments that directly target platelets for the improvement of survival in CRC.

A growing body of evidence reported that lymphocytes could induce apoptosis of tumor cells and were inversely related with tumor proliferation and invasiveness [30, 39]. Therefore, a decreased number of lymphocytes could impede antitumor immune response and further facilitate tumor metastatic potential [4]. Several studies also showed that low-tumor-infiltrating lymphocytes are significantly associated with poor survival in CRC [40, 41]. Accordingly, a systemic review and meta-analysis by Gooden et al. included 10 studies with 3984 CRC patients and indicated that tumor-infiltrating lymphocytes could influence prognosis of CRC [42]. Studies on immunotherapy targeting immune checkpoint (i.e., cytotoxic T-lymphocyte-associated antigen 4 and programmed death 1 receptor) have raised the prospect that the immune system may represent a favorable approach for advancing the treatment of CRC [43, 44]. Further studies are needed to explore the antitumor activity of the host immunity via immunotherapy, especially for subpopulations with lymphopenia.

There were few studies primarily focused on the clinicopathological value of PLR in CRC. In the included studies, we found that the PLR was most frequently elevated in poor clinicopathological characteristics. Accordingly, Azab et al. included patients with stage I to stage IV and the patients were categorized into an equal tertile based on PLR, and results showed that PLR was significantly related with tumor stage (*P* < 0.001) [17]. Ying et al. and Choi et al. showed a significant association between PLR and tumor stage and pT category (*P* < 0.05) [9, 12]. Moreover, Kown et al. reported patients with greater PLR showed an increased likelihood of positive lymph node ratio > 0.2 (*P* = 0.0006) and a lower 5-year OS (*P* = 0.001) [20]. Ozawa et al. also found that patients with a high PLR more frequently suffered obstruction or perforation/penetration and on average had larger tumors compared to those with a low PLR [24]. After pooling all relevant studies, our results also suggested that elevated PLR was associated with poor clinicopathological characteristics (i.e., tumor stage, pT category, and degree of differentiation), suggesting that PLR may be feasible for tumor staging. Thus, according to the above results, greater PLR may reflect an enhanced host inflammatory response to more aggressive tumor biology and higher tumor burden. Future studies should thoroughly evaluate the association between PLR and clinicopathological characteristics for further providing an additional basis for CRC staging.

The definition for the optimal cut-off value of PLR is urgently required and is the main concern for the clinical utility. There is no agreement on the optimal cut-off value, although most included studies defined a cut-off value of 150. It is unclear whether this cut-off value was appropriate for predicting prognosis in all CRC patients. Song et al., Neal et al., and Kwon et al. grouped PLR into three groups (<150, 150–300, and >300), and the results reported that PLR was significantly associated with OS [20, 22, 26]. Ozawa et al., Neofytou et al., Ying et al., and Szkandera et al. applied a receiver operating curve to calculate the optimal cut-off value, but the optimal cut-off value still varied (25.4, 150, 175, and 225, respectively) [12, 23, 24, 28]. The differences of cut-off values may be attributed to the differences of included patients. Indeed, Ozawa et al., Neofytou et al., Ying et al., and Szkandera et al. included stage II CRC, colorectal liver metastasis, stages I–III CRC and II-III colon cancer, respectively. Moreover, Kwon et al. and Song et al. both reported that <15% patients were grouped into the subset of the highest tertile PLR > 300 [20, 26]. Therefore, it was notable that a high cut-off value may lead to the omission of a greater number of patients in clinical practice although it may be more valuable. Further studies are needed to explore whether the optimal cut-off values of PLR differ among different population and then to define the optimal cut-off value of PLR for future individual treatments.

Although previous meta-analyses evaluated the prognostic values of PLR for CRC, the cut-off value of PLR and the association between PLR and clinicopathological characteristics were not assessed [45, 46]. Thus, this study had several obvious advantages. First, our study included more eligible studies, making our results more powerful and robust. Second, our studies assessed the impact of cut-off values on prognostic values. Third, we found that the PLR was most frequently elevated in advanced stage tumor for CRC, and we assessed the prognostic role of PLR in metastasis positive and negative groups using rational and robust subgroups. In addition, we assessed the quality of included studies and then performed subgroup analyses based on the study quality.

There were several limitations in the present study. First, our study was based on the published data. Several HRs with 95% CIs were calculated from available data in the studies that did not provide HRs directly. Second, considerable heterogeneity existed in the present study, and we used a relatively conservative random effects model if there was heterogeneity significant; and, therefore, it may underestimate the prognostic value of PLR in CRC. Although our metaregression analysis did not found significant sources of heterogeneity, the heterogeneity could be also caused by differences in patient characteristics (i.e., age, gender, race, and treatment strategies) and thus heterogeneity could not be completely eliminated. Therefore, further large-scale multicenter studies on homogeneous patients and diagnostic method are required to investigate the prognostic values of PLR in CRC. Moreover, our study did not provide results regarding the optimal cut-off value and whether the cut-off values differed in the assessment of clinicopathological characteristics and prognosis values. In addition, although we performed subgroup analysis based on analysis type in the primary studies (multivariable and univariable analysis), the variables that were included in multivariable analysis or adjustment were different in the primary studies and the number of included studies was also limited, and thus, we could not conducted in-depth subgroup analysis based on the various variables. Further studies are needed to assess the prognostic values of PLR in CRC using optimal multivariable analysis or adjustment.

## 8. Conclusions

Our results indicate that elevated PLR predicted poor prognosis and clinicopathological characteristics in CRC. PLR is a convenient and low-cost approach for the prognostic prediction and individualized treatment for CRC. Future studies are required to identify the optimal cut-off value of PLR and improve the clinical utility of PLR.

## Figures and Tables

**Table 1:** Baseline characteristics and design variables of eligible studies.

Study	Country	Year	Number of patients (M/F)^a^	Sampling time	Age: mean ± SD/ median (range)^b^	Cut-off point	Rate of PLR (*n*/*N*)^c^	Follow-up: mean ± SD/ median (range)^d^	Outcome measured^e^	NOS^f^
Mori	Japan	2015	152 (87/65)	Preoperative	Mean: 66.9; range: 35–89	150	84/152	Median: 20.5 (0.2–62.4)	OS, DFS	6
Song	Korea	2015	177 (83/94)	Before treatment	Median: 52 (25–81)	150	104/177	Median: 3.1 (0.1–33.3)	OS	5
Ozawa	Japan	2015	234 (142/92)	Preoperative	NR	25.4	53/234	Median: 64 (1–173)	OS, DFS, CSS	7
Neal	UK	2015	302 (192/110)	Preoperative	Median: 66 (26–85); mean: 64.8	150	169/302	Median: 29.7 (4–96)	OS, CSS	7
Li	China	2015	110 (58/52)	Preoperative	Mean: 62.9 ± 11.9	162	64/110	Max: 24	OS	6
Choi	Canada	2015	549 (296/253)	Preoperative	Median: 68.7 (28.3–92.6)	295	51/549	Median: 48 (0–124.8)	OS, RFS	8
Azab	USA	2014	580 (273/307)	Before treatment	Mean: 68.62 ± 12.82	147	387/580	Mean: 41.2; median: 40.5	OS, DFS, CSS	7
Neofytou	UK	2014	140 (88/52)	Preoperative	NR	150	58/140	Median: 33 (1–103)	OS, DFS	7
Ying	China	2014	205 (144/61)	Preoperative	NR	176	79/205	NR	OS, RFS, CSS	6
Szkandera	Austria	2014	372 (217/155)	Preoperative	Median: 64 (27–95)	RFS: 176 OS: 225	176 : 217/372; 225 : 164/372	Median: 68 (1–190)	OS, RFS	6
Sun	China	2014	255 (135/120)	Preoperative	Mean: 59.47 ± 12.63	150	120/254	NR	OS, DFS	6
Baranyai	Hungary	2014	CRC: 336 (180/156); mCRC: 118 (80/38)	Preoperative	CRC: 66.9 ± 11; mCRC: 61.0 ± 8.3	300	CRC: 31/336; mCRC: 5/118	Median: 36.1	OS, DFS	6
Son	Korea	2013	624 (368/256)	Preoperative	NR	300	16/624	Median: 42.0 (1–66)	OS, DFS	6
He	China	2013	243 (155/88)	Before chemotherapy	Median: 56 (18–83)	150	130/243	Median: 21.87	OS, DFS	7
Kwon	Korea	2012	200 (123/77)	Preoperative	Median: 64 (26–83)	150	83/200	Median: 33.6	OS	5
Carruthers	UK	2012	115 (75/40)	Pretreatment	Mean: 63.8; range: 32.3–81.1	160	NR	Median: 37.1	OS, RFS, DFS	5
Proctor	UK	2011	374	NR	NR	150	NR	Median: 51 (18–115)	OS, CSS	6

^a^M/F presents the number of males and females, respectively. ^b^The age of patients was summarized as mean with standard deviation or median with range. ^c^The rate of patients with elevated PLR. ^d^The follow-up period was summarized as mean with standard deviation or median with range. ^e^The outcomes assessed (OS, DFS, CSS, or/and RFS) were presented in each included study. ^f^The study quality was assessed with the Newcastle-Ottawa scale criteria. CRC: colorectal cancer; CSS: cancer-specific survival; DFS: disease-free survival; NOS: Newcastle-Ottawa scale criteria; NR: not reported; OS: overall survival; PLR: platelet-to-lymphocyte ratio; RFS: recurrence-free survival SD: standard deviation; UK: United Kingdom; USA: The United States of America.

**Table 2:** Results of subgroup analyses for prognostic significance of platelet-to-lymphocyte ratio.

	Overall survival	Disease-free survival	Cancer-specific survival	Recurrence-free survival
*Overall*	HR = 1.46 (1.23–1.73), *I*^2^ = 60.8%	HR = 1.64 (1.17–2.30), *I*^2^ = 73.5%	HR = 1.30 (1.12–1.50), *I*^2^ = 0.0%	HR = 1.38 (1.09–1.74), *I*^2^ = 20.7%
*Sampling time*				
Preoperative	HR = 1.61 (1.28–2.02), *I*^2^ = 61.4%	HR = 1.78 (1.12–2.83), *I*^2^ = 77.3%	HR = 1.26 (1.04–1.52), *I*^2^ = 33.3%	HR = 1.38 (1.09–1.74), *I*^2^ = 20.7%
*Metastatic status*				
M1	HR = 1.40 (1.23–1.60), *I*^2^ = 26.6%	HR = 1.76 (1.23–2.51), *I*^2^ = 0.0%	/	/
M0	HR = 1.63 (1.15–2.30), *I*^2^ = 68.4%	HR = 1.82 (1.03–3.21), *I*^2^ = 80.7%	HR = 1.75 (0.59–5.17), *I*^2^ = 66.2%	HR = 1.38 (1.09–1.74), *I*^2^ = 20.7%
*Sample size*				
≥250	HR = 1.36 (1.01–1.83), *I*^2^ = 77.3%	HR = 1.46 (0.80–2.64), *I*^2^ = 85.3%	HR = 1.30 (1.11–1.52), *I*^2^ = 0.0%	HR = 1.67 (1.21–2.29), *I*^2^ = 0.0%
<250	HR = 1.53 (1.32–1.77), *I*^2^ = 0.0%	HR = 1.76 (1.35–2.30), *I*^2^ = 0.5%	HR = 1.75 (0.59–5.17), *I*^2^ = 66.2%	HR = 1.11 (0.79–1.56), *I*^2^ = 0.0%
*Cut-off point*				
>150	HR = 1.60 (1.18–2.17), *I*^2^ = 66.7%	HR = 2.03 (0.73–5.62), *I*^2^ = 86.0%	HR = 1.22 (0.93–1.59), *I*^2^ = 0.0%	HR = 1.38 (1.09–1.74), *I*^2^ = 20.7%
≤150	HR = 1.33 (1.08–1.64), *I*^2^ = 58.0%	HR = 1.49 (1.03–2.14), *I*^2^ = 71.0%	HR = 1.34 (1.12–1.59), *I*^2^ = 37.8%	/
*Country*				
Asia	HR = 1.41 (1.22–1.63), *I*^2^ = 45.2%	HR = 1.71 (0.97–3.01), *I*^2^ = 73.9%	HR = 1.75 (0.59–5.17), *I*^2^ = 66.2%	/
Europe & America	HR = 1.46 (1.11–1.91), *I*^2^ = 71.9%	HR = 1.62 (0.99–2.65), *I*^2^ = 79.0%	HR = 1.30 (1.11–1.52), *I*^2^ = 0.0%	HR = 1.49 (1.11–2.00), *I*^2^ = 35.2%
*Study quality*				
≥6	HR = 1.44 (1.18–1.77), *I*^2^ = 65.9%	HR = 1.72 (1.17–2.52), *I*^2^ = 76.7%	HR = 1.30 (1.12–1.51), *I*^2^ = 0.0%	HR = 1.46 (1.14–1.87), *I*^2^ = 0.0%
<6	HR = 1.54 (1.23–1.93), *I*^2^ = 0.0%	/	/	/
*Study analysis type*				
Univariable type	HR = 1.63 (1.29–2.04), *I*^2^ = 57.6%	HR = 2.08 (1.28–3.38), *I*^2^ = 60.6%	HR = 1.31 (1.10–1.56), *I*^2^ = 0.0%	HR = 1.23 (0.60–2.53), *I*^2^ = 63.3%
Multivariable type	HR = 1.32 (1.03–1.69), *I*^2^ = 59.9%	HR = 1.33 (0.91–1.93), *I*^2^ = 66.6%	HR = 1.28 (0.99–1.66), *I*^2^ = 32.7%	HR = 1.38 (1.03–1.85), *I*^2^ = 5.3%

HR: hazard ratio; “/” symbol: no results due to insufficient studies.

**Table 3:** Metaregression analysis exploring sources of heterogeneity.

	Coefficient	Standard error	*P*	Adjusted *R*^2^
*Overall survival*				
Sampling time	0.2797	0.1995	0.18	4.16%
Metastatic status	−0.0362	0.1089	0.744	−11.16%
Sample size	−0.0006	0.0006	0.401	2.35%
Cut-off value	0.0020	0.0016	0.229	23.59%
Country	0.0277	0.1945	0.889	−11.56%
Study quality	−0.0316	0.1196	0.795	−9.52%
*Disease-free survival*				
Sampling time	0.2377	0.4092	0.579	−14.75%
Metastatic status	−0.0380	0.2159	0.865	−19.67%
Sample size	−0.0004	0.0011	0.739	−14.88%
Cut-off value	0.0017	0.0028	0.566	−7.16%
Country	0.0376	0.3659	0.921	−19.62%
Study quality	0.0832	0.2664	0.764	−17.38%

Note: the dependent variable is the lnHR for overall survival or disease-free survival from each study; weights have been assigned according to the estimated variance of the lnHR; cancer-specific survival and recurrence-free survival were not analyzed due to a limited number of studies.

## Additional Points

The corresponding author had full access to all the data and analyses.
